# Anaemia in a phase 2 study of a blood stage falciparum malaria vaccine

**DOI:** 10.1186/1475-2875-10-13

**Published:** 2011-01-19

**Authors:** Ruth D Ellis, Michael P Fay, Issaka Sagara, Alassane Dicko, Kazutoyo Miura, Merepen A Guindo, Aldiouma Guindo, Mahamadou S Sissoko, Ogobara K Doumbo, Dapa Diallo

**Affiliations:** 1 Laboratory of Malaria Immunology and Vaccinology, National Institute of Allergy and Infectious Diseases, National Institutes of Health (NIAID/NIH), Rockville, Maryland, USA; 2 Biostatitistics Research Branch, NIAID/NIH, Bethesda, Maryland, USA; 3 Malaria Research and Training Center, University of Bamako, Mali

## Abstract

**Background:**

A Phase 1-2b study of the blood stage malaria vaccine AMA1-C1/Alhydrogel was conducted in 336 children in Donéguébougou and Bancoumana, Mali. In the Phase 2 portion of the study (n = 300), no impact on parasite density or clinical malaria was seen; however, children who received the study vaccine had a higher frequency of anaemia (defined as haemoglobin < 8.5 g/dL) compared to those who received the comparator vaccine (Hiberix). This effect was one of many tested and was not significant after adjusting for multiple comparisons.

**Methods:**

To further investigate the possible impact of vaccination on anaemia, additional analyses were conducted including patients from the Phase 1 portion of the study and controlling for baseline haemoglobin, haemoglobin types S or C, alpha-thalassaemia, G6PD deficiency, and age. A multiplicative intensity model was used, which generalizes Cox regression to allow for multiple events. Frailty effects for each subject were used to account for correlation of multiple anaemia events within the same subject. Intensity rates were calculated with reference to calendar time instead of time after randomization in order to account for staggered enrollment and seasonal effects of malaria incidence. Associations of anaemia with anti-AMA1 antibody were further explored using a similar analysis.

**Results:**

A strong effect of vaccine on the incidence of anaemia (risk ratio [AMA1-C1 to comparator (Hiberix)]= 2.01, 95% confidence interval [1.26,3.20]) was demonstrated even after adjusting for baseline haemoglobin, haemoglobinopathies, and age, and using more sophisticated statistical models. Anti-AMA1 antibody levels were not associated with this effect.

**Conclusions:**

While these additional analyses show a robust effect of vaccination on anaemia, this is an intensive exploration of secondary results and should, therefore, be interpreted with caution. Possible mechanisms of the apparent adverse effect on haemoglobin of vaccination with AMA1-C1/Alhydrogel and implications for blood stage vaccine development are discussed. The potential impact on malaria-associated anaemia should be closely evaluated in clinical trials of AMA1 and other blood stage vaccines in malaria-exposed populations.

## Background

A vaccine against *Plasmodium falciparum *has long been sought and is a badly needed tool in the fight to eliminate and eradicate malaria. Experiments indicate that parasites and clinical disease can be controlled through passive transfer of antibody [1,2], although specific clinical markers of protection have not yet been elucidated. A partially protective pre-erythrocytic vaccine is currently in Phase 3 trials [3]. However, a "second generation" more highly protective vaccine is needed. Inclusion of blood stage antigens in a multi-stage vaccine would likely lead to higher levels of protection against clinical disease, and would also provide protection against epidemic malaria if pre-erythrocytic immunity is incomplete [4].

AMA1 is one of the leading blood stage vaccine candidates, and was evaluated in a Phase 1-2b trial in malaria-exposed children in Mali [5,6]. This trial evaluated a vaccine combining two allelic forms of AMA1 (AMA1-C1), adjuvanted with Alhydrogel. The vaccine was moderately immunogenic and no overall impact of vaccination on malaria parasitaemia or disease was seen [6]. Allele-specific effects were not demonstrated [7]. As per the analytic plan for the Phase 2 (biologic impact) part of the study, 16 secondary outcomes were examined for data up to day 154. Four of the 16 secondary outcomes examined were related to haemoglobin (Hb); all showed trends towards a negative impact of vaccination with AMA1 relative to the comparator group, with two of these statistically significant at the 0.05 level (mean Hb during clinical malaria, unadjusted p = 0.004, and number of episodes with Hb < 8.5 g/dL, unadjusted p = 0.029), although differences were no longer significant after correction for multiple tests. Additional analyses using all subjects with available data from day 1 until day 364 showed significance of Hb effects: both minimum Hb [p = 0.0121] and mean Hb during clinical malaria events [p = 0.0044] were significantly lower in the AMA1 group, and the incidence of Hb < 8.5 g/dL [p = 0.0096] was more frequent in the AMA1 group. An extended follow-up period from November/December 2007 until January 2008 was added due to concern about the possible impact on anaemia events, and an imbalance in serious adverse events related to malaria. However, no significant differences in Hb endpoints were found using only the extended follow-up data [6].

This paper describes further analyses of the differences between the AMA1-C1 vaccine and the control (Hiberix) groups in the study described in [6]. Additional analyses focused on the repeated incidence of anaemia and explored possible confounders or mechanisms of action of the effect of vaccination on Hb. Variables included were: baseline Hb, as lower Hb at the start of the malaria transmission season has been shown to be associated with anaemia during clinical malaria [8]; Hb S, C, and alpha-thalassaemia, as variant Hb is known to be protective against severe malaria and Hb S has been shown to delay time to first clinical malaria event [9-12], and these variables could also affect the risk of anaemia. Similarly, G6PD status is a potential confounder for anaemia [13] and was included in the model. Age is a risk factor for development of anaemia, with younger children more vulnerable [14], therefore age was added. Data on 24 subjects from the Phase 1 part of the study was also included (see [5]).

## Methods

### Description of study

The trial was conducted under a clinical protocol reviewed by the Institutional Review Board of NIAID/NIH, and by the Ethics Committee of the University of Bamako, Faculty of Medicine, Pharmacy, and Odonto-Stomatology. Details on the study design and results of primary and secondary outcomes have been published [5,6]. Briefly, in the dose-escalating Phase 1 part of the study, 36 2-3 year old children were randomized 1:1:1 to receive 20 μg AMA1-C1/Alhydrogel, 80 μg AMA1-C1/Alhydrogel, or the comparator vaccine (Hiberix, Hemophilus influenza B), with two doses given at a four-week interval. In the Phase 2 part of the study, 300 children were randomized 1:1 to receive either 80 μg AMA1-C1/Alhydrogel or the comparator. Children were followed both actively and passively to study day 154, with parasite density (by malaria smears) and haemoglobin (by Hemocue) measured monthly and at sick visits through the malaria transmission season. Hb was also measured as part of complete blood counts at several time points during the study. Any time two measurements of Hb were done on the same day for the same subject, the minimum of the two was taken for the response. Screening Hb < 8.5 g/dL was an exclusion criterion, although a few children had baseline Hb lower than this on the day of enrollment since results of Hb were not known prior to vaccination on Day 0.

Anti-AMA1 antibody levels were determined using a standardized ELISA as previously described [15]. Hb and alpha-thalassaemia typing, and G6PD deficiency were determined as previously described [13] from specimens obtained at the start of the second transmission season, when children in the study were re-enrolled for additional follow up.

### Statistical methods

#### Data selection

As this was a safety analysis, all children with data available from the Phase 1 and Phase 2 studies were included in the analysis (intent to treat), except that children in the Phase 1 part of the study who received the low dose (20 μg AMA1-C1) were excluded. Additionally, children missing all Hb values after baseline, and children missing Hb type, alpha-thalassaemia, or G6PD were excluded from the primary analysis. For the primary analysis only the first 154 days for each subject were included, since that was the period of intensive follow-up during which Hb was measured frequently.

#### Primary model

The primary analysis was an Anderson-Gill-type multiplicative intensity model on the anaemia (Hb < 8.5 g/dL) incidence [16]. This model is similar to a Poisson model on the rate of anaemia, but has several enhancements. First, it is a time to event analysis (similar to Cox regression but allowing multiple events), so that it adjusts for the malaria season by allowing different intensity function (analogous to the hazard function in Cox regression) at each calendar time at risk. Modeling the "baseline" intensity by calendar time instead of time since randomization, as is usually done, is preferable for these data because children entered the study in a staggered fashion through the early malaria transmission season and there is a strong seasonal effect on malaria in Mali. A further enhancement is that a random effect (i.e., a frailty effect) is added for each subject in order to adjust for correlation of events within a subject. The gamma distribution was used to model this variability of the subjects' intensity rates (i.e., to model the frailty) (see [16], section 9.5).

Baseline Hb is an important covariate in the frequency of anaemia in this population [8]. Standard methods either use a categorical expression of this variable (e.g., 2 groups: Hb < 10 or Hb > = 10) or treat it as continuous. Here the functional form for this covariate was examined by fitting two separate Poisson models on anaemia incidence rate using all the other covariates within each vaccine arm. The models included effects for sex, age at baseline (treated as a continuous variable), G6PD deficiency (which is binary, equal to 1 for hemizygous males and homozygous females and 0 for all others, because heterozygous females are not expected to have a strong effect), HbS (equals 1 for Hb type = AS or SC, 0 otherwise), HbC (equals 1 for Hb type = AC or SC, 0 otherwise), and alpha-thalassaemia (categorical variable with 2 degrees of freedom, representing the three classes: wild-type, heterozygous, and homozygous). There were no individuals with the CC or SS phenotype; individuals with the SC phenotype contribute to both the HbS and HbC variable. Residuals were then plotted against baseline Hb and a nonparametric regression was used to plot the functional form. Residuals from the Poisson rate model were used since it is not straightforward how to derive a single residual per subject from the multiplicative intensity model, and the Poisson model should give a similar functional form. An offset for the number of days at risk was used in the Poisson regression, calculated for each subject using the number of days from entering the study until the last Hb measurement was taken. The offset is the standard way that rates are modelled using Poisson regression, allowing us to correctly account for the Poisson-like variance of the counts, while modelling relative risks in terms of the rates not the counts (see for example, [17]). In the residual plot from these models, a nonparametric smoother was then used (the lowess function in R using the default span, [18]) to try and see the functional form that baseline Hb had on the rate of anaemia. From these residual plots, a simple functional form for baseline Hb was defined to be included in all subsequent models. A multiplicative intensity model was then run to see if there was an effect of vaccine group after controlling for baseline Hb as described, as well as the other covariates (sex, age, G6PD, HbS, HbC, alpha-thalassaemia).

### Additional analyses

**Figure 1:**
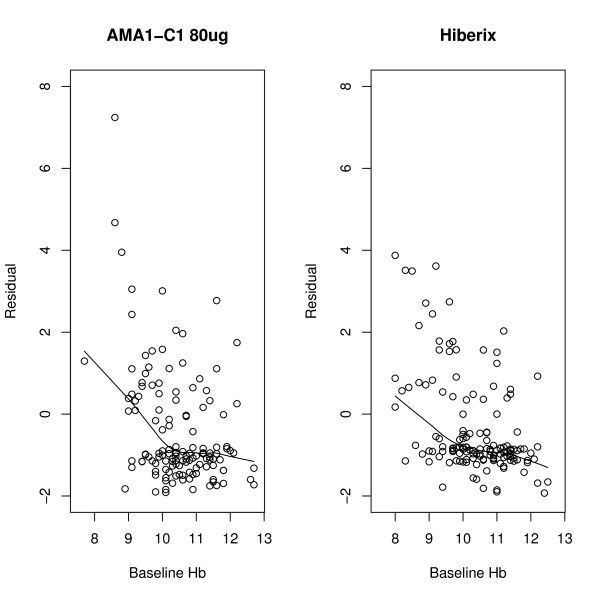
**Plot of Residual from Poisson Regression**. Lines are lowess smooths and motivate the functional form of the effect of baseline Hgb on the incidence rate of Hb < 8.5.

The relationship of vaccine to maximum severity of anaemia adverse events was evaluated using exact Wilcoxon-Mann-Whitney (WMW) tests, where the adverse events were graded according to the age at which they occurred, and aged-stratified grading scales were based on data from previous studies in children in Mali [8] (Table 1). Since the primary analysis continued to show a vaccine effect on anaemia rate, the relationship between minimum Hb and antibody levels at day 42 was explored. The lowess smoother was used in a manner similar to that described above for Figure 1. To test for relationships in non-ordered variables (e.g., Hb type), Fisher's exact test was used. To see if the anaemia effects were substantially driven by unscheduled or sick visits, separate analyses were performed using only scheduled or unscheduled visits. Analyses were done in R (version 2.11.1) using the survival package (version 2.35-8) for the primary analysis and the coin package (version 1.0-11) for the exact WMW tests [19].

**Table 1:** Grading scale for haemoglobin related adverse events (based on data from Dicko, Klion et al [8]).

Age	Normal	Grade 1	Grade 2	Grade 3	Grade 4
1-2 years old	9-10.5	8.0-8.9	7.0-7.9	6.0-6.9	< 6.0
3 years old	10.0-11.5	9.0-9.9	8.0-8.9	7.0-7.9	< 7.0
4 years old	10.5-11.5	9.4-10.4	8.3-9.3	7.0-8.2	< 7.0

All values are g/dL.

## Results

### Description of subjects for primary analysis

Two (2) children withdrew from the study immediately after first vaccination and, therefore, did not have any Hb measurements other than baseline, and were not included. Of the remaining 322 children who received either 80 μg AMA1-C1 or the comparator (161 in each group), Hb type was obtained for 276 (134 who received the study vaccine and 142 who received the comparator), and alpha-thalassaemia and G6PD status were obtained for 275 children (134 who received the study vaccine and 141 who received the comparator). For the primary analysis, data were available for 133 subjects who received the study vaccine (12 in Phase 1, 121 in Phase 2), and 141 who received the comparator (11 in Phase 1, 130 in Phase 2). The primary data had a fairly even distribution of the Hb types (Fisher's exact test, p = 0.586), and of the G6PD status (Fisher's exact test p = 0.420), but alpha-thalassaemia was less evenly distributed (Fisher's exact test p = 0.040) (see Tables 2, 3, and 4).

**Table 2:** Frequency of hemoglobin variants and G6PD deficiency in subjects in primary analysis - Haemoglobin types

*Vaccine received*	*AC*	*AS*	*SC*	*AA*
AMA1-C1	12	18	2	101
Hiberix	16	18	0	107

AC = heterozygous for hemoglobin C, AS = sickle trait, SC = hemoglobins S and C, AA = wild type

**Table 3:** Frequency of hemoglobin variants and G6PD deficiency in subjects in primary analysis - Alpha-thalassaemia

*Vaccine received*	*HE*	*HO*	*WT*
AMA1-C1	34	10	89
Hiberix	35	2	104

HE = heterozygous, HO = homozygous, WT = wild type

**Table 4:** Frequency of hemoglobin variants and G6PD deficiency in subjects in primary analysis - G6PD

*Vaccine received*	*A-*	*A-/-*	A*	*A+*
AMA1-C1	6	3	4	120
Hiberix	3	1	7	130

A- = hemizygous males, A-/-homozygous females, A* = heterozygous females, A+ = wild type

Baseline Hb was similar in the two groups (AMA1 group mean = 10.47, comparator mean = 10.35, t-test p = 0.311); however, when baseline Hb was examined in terms of adverse events graded according to the age-stratified grading scales, an uneven distribution of anaemia events in the groups at baseline was seen (see Table 5, Exact Wilcoxon-Mann-Whitney test p = 0.002). There were significantly higher grade anaemia events by this scale in the comparator (Hiberix) group than in the AMA1-C1 group at baseline.

**Table 5:** Anaemia events at baseline, according to age-stratified grading scale (see Table 1)

	*AMA1-C1*	*Hiberix*
Grade 0	120	107
Grade 1	11	29
Grade 2	2	5

### Primary analysis

Residuals for Hb are shown in Figure 1. From this figure, two new variables were created that act on the anaemia incidence rate: one variable represents a linear function for baseline Hb < 10 and a second variable represents a possibly different linear function for baseline Hb > = 10.

The results of the multiplicative intensity model are given in Table 6. The AMA1-C1 group had a risk of anaemia twice that of the comparator vaccine, with a risk ratio of 2.01 (95% Confidence Interval 1.26, 3.20, p = 0.0035). The other significant effects were for subjects with baseline hg < 10, with each g/dL below 10 increasing the risk of subsequent anaemia by a factor of 4.26 (95% CI 2.72, 6.69; p < 0.0001), consistent with previous results in Mali [8], and age at time of anaemia event, with the risk ratio reduced by a factor of 0.45 for each year older (95% CI 0.29, 0.70; p = 0.0004).

**Table 6:** Results of Primary Multiplicative Incidence Model on Risk of Hg < 8.5 g/dL

*Variable*	*Risk Ratio*	*Two-sided p-value*
	*(95% CI)*	*(NS > 0.25)*
Vaccination with 80ug	2.01 (1.26, 3.20)	0.0035
AMA1-C1		
Age at time of anaemia event*	0.45 (0.29, 0.70)	0.0004
Baseline Hg > = 10 g/dL	0.78 (0.52, 1.17)	NS
Baseline Hg < 10 g/dL	4.26 (2.72, 6.69)	< 0.0001
Male Sex	0.74 (0.46, 1.17)	NS
HbS	0.83 (0.42, 1.61)	NS
HbC	0.96 (0.43, 2.15)	NS
G6PD deficiency	2.27 (0.85, 6.04)	NS
α-thalassaemia heterozygote	0.71 (0.41, 1.23)	NS
α-thalassaemia homozygote	0.41 (0.12, 1.45)	NS

* Risk ratio is reduced by a factor of 0.452 for each year older

### Modifications to primary analysis

Since the additional added variables (sex, Hb type, and G6PD status) did not appear to be important, a simpler model without the non-significant variables was also used, enabling the inclusion of more subjects (161 in each vaccine group). A similar estimate of the risk ratio was obtained for AMA1-C1 group: Risk Ratio = 1.71, 95% CI 1.09, 2.66, p = 0.019. A simpler Poisson model, still including all the variables in Table 6 (and consequently using the same set of subjects as in the primary analysis) also gave similar results (rate ratio 2.22, 95% CI 1.45, 3.40; p = 0.0003).

**Figure 2:**
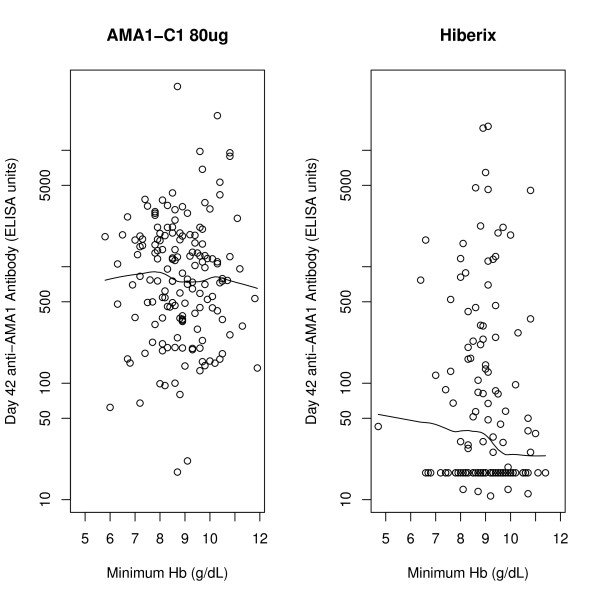
**Day 42 anti-AMA1 antibody plotted against minimum Hb for AMA1 and comparator groups, with nonparametric fits**.

**Figure 3:**
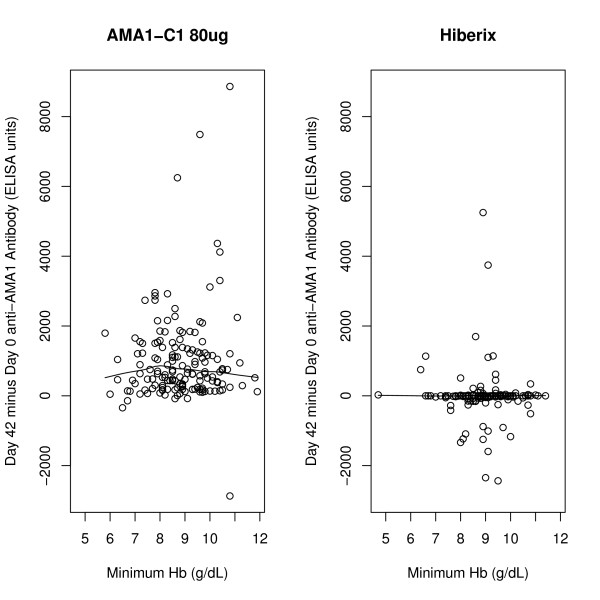
**Day 42-Day 0 anti-AMA1 antibody plotted against minimum Hb for AMA1 and comparator groups, with nonparametric fits**.

Since vaccination with AMA1-C1 had a significant effect on the level of anti-AMA1 antibody (see Figure three in Ref [6], antibody levels were examined for a possible relationship to anaemia incidence. The vaccine was given at days 0 and 28 and had peak values of anti-AMA1 ELISA values at vaccine day 42. Using the primary analysis described above, except only including anaemia events occurring between day 42 and day 154 (rather than between day 0 and day 154), an effect of vaccine was still seen (RR = 2.09, p = 0.010). Using the same data set, the variable for vaccine group was replaced with the average of the 3D7 and FVO anti-AMA1 antibody levels within each subject at day 42. If the effect of the vaccine was mediated through the level of antibody, this would be expected to be a significant effect; however, no effect was seen (HR for an increase in day 42 antibody by 1000 ELISA units = 0.96, 95% CI [0.84, 1.10], p = 0.59). Similarly, no significant effect of day 42 antibody was seen when separate analyses were performed within each vaccine group, nor when log transformed antibody units were used. To see the lack of effect in a different manner, in Figure 2 the average day 42 anti-AMA1 antibody by minimum Hb for both vaccine groups was plotted and nonparametric fits were drawn. No obvious relationship of anti-AMA1 antibody to minimum Hb was observed; although there is an apparent trend for higher day 42 anti-AMA1 antibody to lead to lower minimum Hb in the Hiberix group, this effect is not significant (p = 0.209 by linear regression on log transformed antibody values). A similar plot for the change in anti-AMA1 antibody (day 42-day 0) also showed no effects (Figure 3).

### Additional analyses

Table 7 shows the maximum severity anaemia adverse events occurring after vaccination in the AMA1-C1 and comparator groups. Higher grade anaemia events occurred in the AMA1 group, with borderline significance of this effect (exact Wilcoxon-Mann-Whitney p = 0.051). Finally, to see if the anaemia effects seen were manifested primarily during the unscheduled visits, the primary analysis was repeated twice more, first using only Hb measured on scheduled visits and then using only Hb measured at sick or unscheduled visits (presumably often due to malaria). Both results were similar to the primary analysis (RR [AMA1-C1 to comparator] from scheduled = 2.10 and from unscheduled = 2.24).

**Table 7:** Maximum Anaemia Adverse Event Grade at any time after vaccination

Severity	AMA1-C1 (n = 161)		Hiberix (n = 161)	
	n	% (95% CI)	n	% (95% CI)
Grade 0	32	19.9 (14.0-26.9)	40	24.8 (18.4-32.3)
Grade 1	50	31.1 (24.0-38.8)	58	36.0 (28.6-44.0)
Grade 2	48	29.8 (22.9-37.5)	43	26.7 (20.1-34.2)
Grade 3	24	14.9 (9.8-21.4)	17	10.6 (6.3-16.4)
Grade 4	7	4.3 (1.8-8.8)	3	1.9 (0.4-5.3)

## Discussion

This intensive exploration of the relationship of AMA1 vaccine with anaemia was motivated by secondary results uncorrected for multiple comparisons, so results should be interpreted not as a confirmatory study but as the continuation of an exploratory study. Nevertheless, despite these cautions on over-interpretation, effects were still seen when more subjects were included, more potentially confounding variables were included, and more statistically sophisticated analyses were performed. The strengthening of the effect after inclusion of baseline haemoglobin may be because of the uneven randomization at baseline, i.e. children enrolled in the comparator group had lower baseline haemoglobin and more anaemia, thus partially masking the strength of the association of vaccination of AMA1 and anaemia in the initial analysis. The imbalance in anaemia was seen in higher grade as well as lower grade events, although numbers were small and this difference was not statistically significant. Clearly the possible impact on anaemia should be closely evaluated in future clinical trials of AMA1 in malaria-exposed populations.

In theorizing about the possible causes of this apparent anaemia effect, the first possibility that should be considered is that the comparator Hemophilus influenza B vaccine (Hiberix) was somehow protective against anaemia, perhaps through a general improvement in health status. However, this vaccine primarily protects against invasive Hib disease in the first year of life, and also would be expected to exert a level of herd immunity to non-vaccinated children in the village [20]. No previous studies of this class of vaccines which showed protection from anaemia were found in a literature search, and a protective effect of Hiberix is unlikely.

If the increase in anaemia in the group receiving the AMA1 vaccine is a true effect of vaccination, what possible mechanisms could explain this? Experimental *falciparum *challenge studies in *Aotus *monkeys have shown profound anaemia in some cases, and partial control of infection resulting in persistent low grade infection has been postulated to be a possible mechanism for this effect [21]. In the Phase 2 trial parasite densities were similar between the vaccinated and control groups, and time to first event (malaria infection, and fever at varying levels of parasitaemia) was similar between the groups. Thus partial control of parasitaemia appears to be an unlikely explanation in this case.

The aetiology of malaria anaemia is not well understood [22,23]. Three mechanisms are thought to interact to cause anaemia: lysis of infected and uninfected red blood cells primarily during acute infection, sequestration (also occurring during acute infection), and bone marrow suppression. The nadir in haemoglobin typically occurs 4-5 days after treatment, and recovery from anaemia can be prolonged. No difference in clinical evidence of haemolysis (such as jaundice or splenomegaly) was seen between the groups in this study, although these were not study endpoints and it is possible that a difference occurred but was not detected. Coombs tests, haptoglobin, and reticulocyte counts were not done. Sequestration is mediated by adherence factors, possibly related to antibody. No association was seen here between peak (day 42) or induced (day 42-day 0) anti-AMA1 antibody levels and minimum hemoglobin. Although there is no evidence showing a qualitative difference in vaccine-induced anti-AMA1 antibody compared to the infection-induced antibody judged by surface plasmon resonance technique or in vitro growth inhibition assay (unpublished observation), there may be some qualitative difference which these assays are not able to detect, possibly contributing to increased lysis or adherence. Pro-inflammatory cytokines are also thought to play a role in malaria anaemia, with excessive production of Th1 cytokines thought to be beneficial during the early stages of a plasmodial infection and Th2 cytokines likely to be protective in chronic infection or in the recovery period [24,25]. The alum adjuvant used in this vaccine induces a Th2 biased response, and it is theoretically possible that when given with a blood stage antigen the immune response is adversely shifted, although how this would act remotely from the time of vaccination is not clear.

Other efficacy field trials have been conducted with blood stage vaccines. A Phase 2b trial in Kenyan children of FMP1 (MSP1) adjuvanted with AS02 showed no overall protection despite high levels of induced antibody [26]. An imbalance was seen in the number of cases of transient low haemoglobin (9 in 200 vaccinees vs. 2 in 200 controls) but no significant difference in the risk of Hb < 8.0 g/dL was seen and time to first episode of Hb < 8.0 did not differ between the groups, although there was a trend towards an increase in the vaccine group (Hazard ratio 1.53 (95% CI 0.90, 2.59, p = 0.11). Results of a Phase2b trial in Malian children of FMP2 (AMA1) adjuvanted with AS02 also showed no overall protection despite high levels of antibody, although strain specific effects were seen in the first year (5^th ^Multilateral Initiative on Malaria, November, 2009; and 2009 annual meeting of the American Society of Tropical Medicine and Hygiene). No imbalance in anaemia was seen, although these results are not yet published and additional analysis is pending. A Phase 2b trial in Malian children of MSP3 adjuvanted with alum is ongoing (ClinicalTrials.gov Identifier: NCT00652275), and a Phase 2b trial of a GLURP/MSP3 fusion protein also adjuvanted with alum is planned (5^th ^MIM Pan-African Malaria Conference, 2009, Symposium 14).

While it is possible that the association of anaemia with vaccination with AMA1-C1/Alhydrogel in this study is an artifact, and no mechanism is apparent, the robustness of these findings and the imbalance in anaemia seen in one other field trial of a blood stage vaccine [26] are cause for concern. The impact of vaccination on anaemia outcomes should be closely examined, particularly for field trials of blood stage vaccines in malaria-exposed children.

## Competing interests

The authors declare that they have no competing interests.

## Authors' contributions

RDE designed the study, coordinated study execution and analysis, and wrote the paper. MPF designed the study, analysed the data, and co-wrote the paper. IS and AD conducted the field study. KM performed the immunologic analysis. MAG and AG performed clinical laboratory analyses. MSS conducted the field study and was responsible for the study database. OKD and DD designed the study and supervised conduct in the field. All authors reviewed the manuscript and approved the final draft.
